# 四种预后评分系统在早期无症状慢性淋巴细胞白血病患者中评估价值比较

**DOI:** 10.3760/cma.j.issn.0253-2727.2021.10.007

**Published:** 2021-10

**Authors:** 业钦 沙, 晖 沈, 微 吴, 奕 夏, 祎 缪, 蕾 曹, 莉 王, 磊 范, 卫 徐, 建勇 李, 华渊 朱

**Affiliations:** 1 南京医科大学第一附属医院，江苏省人民医院血液科 210029 Department of Hematology, the First Affiliated Hospital of Nanjing Medical University, Jiangsu Province Hospital, Nanjing 210029, China; 2 江苏省人民医院浦口分院血液科，浦口慢淋中心，南京 211800 Pukou CLL Center, Pukou Division of Jiangsu Province Hospital, Nanjing 211800, China

**Keywords:** 白血病，淋巴细胞，慢性, 预后, Leukemia, lymphocytic, chronic, Prognosis

## Abstract

**目的:**

比较四种预后评分系统预测Binet A期中国慢性淋巴细胞白血病（CLL）患者诊断到治疗时间（TTFT）的预后评估价值。

**方法:**

回顾性分析南京医科大学第一附属医院（浦口慢淋中心）2009年6月至2020年1月诊断的110例Binet A期CLL患者的基线临床资料，采用无症状早期CLL国际预后评分（IPS-E）、CLL国际预后指数评分（CLL-IPI）、CLL1预后评分模型（CLL1-PM）与巴塞罗那预后评分（Barcelona-Brno）模型对患者进行危险度分层和预后评估。

**结果:**

110例Binet A期CLL患者中位年龄58（25～84）岁，中位随访时间35（4～189）个月，其中57例（51.8％）患者因病情进展达到治疗指征启动治疗。对患者年龄、Rai分期、淋巴细胞绝对计数（ALC）、淋巴结大小、淋巴细胞倍增时间（LDT）、β_2_-微球蛋白、IGHV突变状态、TP53缺失和（或）突变、11q缺失等9个因素进行Log-rank检验，其中RaiⅠ～Ⅲ期、ALC>15×10^9^/L、淋巴结≥1 cm、β_2_-微球蛋白>3.5 mg/L、IGHV无突变、TP53缺失和（或）突变、11q缺失是影响TTFT的独立危险因素。使用一致性指数（C-index）和赤池信息准则（AIC）对上述模型进行预后评估效能评价，其中CLL1-PM C-index＝0.736，AIC＝777；CLL-IPI C-index＝0.722，AIC＝933；IPS-E C-index＝0.683，AIC＝1004；Barcelona-Brno C-index＝0.663，AIC＝986。

**结论:**

四种预后评分模型均具有预测TTFT的效能。IPS-E因纳入指标的临床可及性高，价格较低，可作为指导临床检测的理想工具。对于完善FISH及二代测序检查的患者，使用CLL-IPI或CLL1-PM可进行更为全面的预后评价。

## Abstract (en)

**Objective:**

To compare the prognostic value of four prognostic models in predicting time to first treatment（TTFT）in patients with Binet A Chinese chronic lymphocytic leukemia（CLL）.

**Methods:**

This retrospective analysis included one hundred and ten patients with Binet A CLL, initially diagnosed in the First Affiliated Hospital of Nanjing Medical University（Pukou CLL center）from June 2009 to January 2020. Risk stratification was conducted according to IPS-E, CLL-IPI, CLL1-PM, and Barcelona-Brno prognostic models.

**Results:**

Among 110 patients with Binet A CLL patients, the median age was 58（25–84）years. The median follow-up time was 35（4–189）months, and 57（51.8％）patients met the indication for treatment due to symptomatic disease progression during follow-up. Log-rank analysis of nine variables was conducted involving age, Rai stage, absolute lymphocyte count（ALC）, lymph node size, lymphocyte doubling time（LDT）, β_2_-Microglobulin, IGHV status, TP53, and Del（11q）. Additionally, Rai Ⅰ–Ⅲ, ALC>15×10^9^/L, palpable lymph node size ≥1cm, β_2_-Microglobulin >3.5 mg/L, unmutated IGHV, TP53 mutation or deletion, and 11q deletion were independent risk factors of TTFT. Predictive value of each model was assessed by Harrel C-index and Akaike information criterion（AIC）with CLL1-PM（C-index＝0.736, AIC＝777）, followed by CLL-IPI（C-index＝0.722, AIC＝933）, IPS-E（C-index＝0.683, AIC＝1004）, and Barcelona-Brno prognostic model （C-index＝0.663, AIC＝986）.

**Conclusion:**

All four prognostic models effectively predicted TTFT. IPS-E might be an ideal model to guide clinical surveillance because of its easy accessibility and low expenses in routine clinical practice. Therefore, for patients receiving fluorescence in situ hybridization（FISH）and next-generation sequencing（NGS）examination at diagnosis, CLL-IPI or CLL1-PM could be applied to evaluate their prognosis more comprehensively.

慢性淋巴细胞白血病（CLL）是西方国家最常见的白血病类型，而在亚洲人群中发病率较低[1]–[2]。不同CLL患者的预后呈高度异质性，约1/3患者确诊后可采取“观察等待”策略而终生无需治疗，部分患者会在诊断后不同时间窗内出现疾病进展而需要治疗[3]–[4]，因此患者确诊后需要进行预后评估从而选择不同的临床随访策略。研究发现多种分子与临床预后指标能够提示患者需要接受治疗的可能性，并基于这些指标提出了各种预后模型预测诊断到治疗时间（TTFT）。考虑到CLL的异质性，对现有模型进行系统评价，综合选择能够广泛适用于CLL患者且临床可及性较高的预后评分模型具有重要的临床意义。

既往研究中，研究者提出了多种预后模型预测TTFT。CLL国际预后指数（CLL-IPI）评分系统被临床广泛运用，其纳入了包括TP53异常状态、免疫球蛋白重链可变区（IGHV）突变状态、血清β_2_-微球蛋白水平、临床分期和年龄等五项独立预后因素，首先被证实可用于预测CLL患者的总生存（OS）[5]。该模型亦被进一步证实能够有效地预测患者的TTFT[6]。Delgado等[7]对CLL-IPI模型进行简化并提出了巴塞罗那预后评分（Barcelona-Brno）模型，该模型仅包括IGHV无突变状态与17p和（或）11q缺失两个独立危险因素。最近有研究针对早期无症状CLL患者建立了国际预后评分指数（IPS-E），其中IGHV无突变、淋巴细胞绝对计数（ALC）>15×10^9^/L、出现可触及的淋巴结被证明是TTFT的独立高危因素[8]–[9]。另外，德国慢性淋巴细胞白血病工作组基于CLL1临床研究针对新诊断的Binet A期患者建立了预后模型（CLL1-PM），17p缺失［del（17p）］、IGHV无突变、11q缺失［del（11q）］、β_2_-微球蛋白>3.5 mg/L、淋巴细胞倍增时间（LDT）<12个月以及年龄>60岁是其独立危险因素[9]。

本研究纳入在我中心诊断的110例早期无症状CLL患者，率先对近年来基于大宗队列提出的四种预后评分模型（IPS-E、CLL-IPI、CLL1-PM和Barcelona-Brno）的效能进行系统性比较与评价。四种预后评分模型均基于西方人群构建，考虑到CLL的生物学在西方人群和亚洲人群间存在差异，在中国CLL患者中对上述预后模型进行系统性验证与评价有利于更好地指导临床选用合适的预后模型，具有重要价值。

## 病例与方法

1. 病例：纳入2009年6月至2020年6月在南京医科大学第一附属医院（浦口慢淋中心）诊断且无治疗指征的Binet A期CLL患者110例。所有患者均进行外周血或骨髓细胞形态学和流式细胞术免疫表型检测，诊断标准与疾病分期严格参照国际CLL工作组（iwCLL）2018指南[4]。所有纳入的患者在诊断后每半年于我院门诊进行临床随访，随访截止时间为2020年10月1日。随访方式主要为查阅门诊及住院病历、电话随访。本研究终点为TTFT，具体定义为患者诊断CLL至因病情进展开始接受治疗的时间间隔。我们纳入患者诊断时前述四种预后模型中涉及的所有指标，包括：①临床信息：年龄、CLL Binet与Rai分期；②血液常规检查指标：ALC、LDT；③生化指标：β_2_-微球蛋白；④分子遗传学指标：del（17p）与TP53突变状态、del（11q）；⑤IGHV突变状态等。运用4种预后模型对本研究队列中的患者进行风险分层，根据TTFT评估不同预后模型的效能。

2. 统计学处理：采用SPSS 25.0软件与R 4.0.2软件进行统计学分析，采用Log-rank检验进行单因素分析，并运用Kaplan-Meier法绘制生存曲线。使用一致性指数（C-index）和赤池信息准则（AIC）评价不同预后模型的预测效能。C-index为1.0提示最佳预测效能，而C-index为0.5则提示完全无预测效能。AIC用于描述预后模型的准确性，AIC值低提示模型准确性高。双侧*P*<0.05表示差异有统计学意义。

## 结果

1. 一般资料：110例诊断时尚无治疗指征的Binet A期CLL患者中，男68例（61.8％），女42例（38.2％），中位年龄58（25～84）岁。中位随访时间35（4～189）个月，57例（51.8％）患者在随访期间由于病情进展出现治疗指征，患者的其他临床特征见表1。

**表1:** 110例慢性淋巴细胞白血病患者诊断到治疗时间（TTFT）的单因素分析

因素	例（％）	中位TTFT（月）	预计5年内未治疗率（％）	TTFT	
				*HR*（95％ *CI*）	*P*值
年龄					
≤60岁	63（57.3）	40	47.3		
>60岁	47（42.7）	66	50.4	0.791（0.469～1.333）	0.383
Rai分期					
0期	40（36.4）	未达到	66.0		
Ⅰ～Ⅲ期	70（63.6）	31	39.5	2.467（1.458～4.173）	0.003
ALC					
≤15×10^9^/L	47（44.8）	未达到	60.4		
>15×10^9^/L	58（55.2）	32	40.5	1.818（1.061～3.115）	0.032
淋巴结大小					
<1 cm，不可触及	51（50.0）	未达到	57.8		
≥1 cm，可触及	51（50.0）	23	42.2	1.875（1.078～3.263）	0.023
LDT					
≥12个月	56（51.9）	未达到	58.8		
<12个月	52（48.1）	34	37.8	1.578（0.928～2.685）	0.087
β_2_-微球蛋白					
≤3.5mg/L	80（80.8）	未达到	58.1		
>3.5mg/L	19（19.2）	16	10.5	3.780（1.660～8.700）	<0.001
IGHV突变状态					
突变	80（73.4）	100	59.0		
无突变	29（26.6）	15	23.0	3.200（1.589～6.444）	<0.001
TP53状态					
正常	93（88.6）	66	53.0		
缺失和（或）突变	12（11.4）	12	16.7	3.278（1.109～9.692）	<0.001
del（11q）					
无	80（90.9）	66	53.6		
有	8（9.1）	9	12.5	3.383（0.903～12.678）	0.001

注：ALC：淋巴细胞绝对计数；LDT：淋巴细胞倍增时间；IGHV：免疫球蛋白重链可变区

2. 影响患者TTFT的预后因素分析：单因素分析结果提示，Rai分期Ⅰ～Ⅲ期、ALC>15×10^9^/L、可触及淋巴结≥1 cm、β_2_-微球蛋白>3.5 mg/L、IGHV无突变、TP53缺失和（或）突变、del（11q）是影响患者TTFT的不良预后因素；而年龄>60岁与LDT<12个月与TTFT的不良预后无显著相关性（表1）。

3. 使用四种预后模型进行预后危险度分层：我们根据IPS-E、CLL-IPI、CLL1-PM和Barcelona-Brno模型对本研究纳入的110例Binet A期患者进行危险度分层（表2）。其中100例CLL患者可进行IPS-E模型评分，并分为低危组（23例）、中危组（36例）、高危组（41例），其中位TTFT分别为未达到、51个月和17个月，预计5年内未治疗概率分别为77.9％、49.3％和35.2％，各组无治疗生存的差异有统计学意义（*P*<0.001）（图1A）。77例患者可进行CLL1-PM模型评分，并分为低危组（38例）、中危组（22例）、高危组（10例）、极高危组（7例），其中位TTFT分别为未达到、51个月、7个月与12个月，预计5年内未治疗概率分别为73.8％、41.0％、20.0％与0，各组无治疗生存的差异有统计学意义（*P*<0.001）（图1B）；组间比较示高危组与极高危组中位TTFT的差异无统计学意义（*P*＝0.425），其余各组间差异有统计学意义（表2）。93例患者可应用CLL-IPI模型评分，分为低危组（45例）、中危组（30例）、高危组（10例）、极高危组（8例），其中位TTFT分别为未达到、31个月、23个月与7个月；预计5年内未治疗概率分别为66.4％、45.2％、20.0％与0；各组无治疗生存的差异有统计学意义（*P*<0.001）（图1C）。98例CLL患者可应用Barcelona-Brno模型评分，分为低危组（69例）、中危组（15例）、高危组（14例），其中位TTFT分别为未达到、31个月和9个月，预计5年内未治疗概率分别为60.5％、40.0％和7.1％；各组无治疗生存的差异有统计学意义（*P*<0.001）（图1D）。

**表2:** 110例慢性淋巴细胞白血病患者IPS-E、CLL1-PM、CLL-IPI和Barcelona-Brno模型危险度分层及无治疗生存比较

预后模型	例数（％）	启动治疗例数（％）	TTFT［月，*M*（95％ *CI*）］	无治疗生存	
				对照组	*P*值
IPS-E					
低危组	23（23.0）	6（26.1）	未达到		
中危组	36（36.0）	17（47.2）	未达到	低危组	0.077
高危组	41（41.0）	27（65.9）	17.0（9.5～24.5）	中危组	0.019
CLL1-PM					
低危组	38（49.3）	11（28.9）	未达到		
中危组	22（28.6）	12（54.5）	51.0（18.0～84.0）	低危组	0.028
高危组	10（13.0）	8（80.0）	7.0（2.4～11.6）	中危组	0.032
极高危组	7（9.1）	7（100.0）	12.0（1.7～22.3）	高危组	0.425
CLL-IPI					
低危组	45（48.4）	15（30.3）	未达到		
中危组	30（32.2）	16（53.3）	未达到	低危组	0.037
高危组	10（10.8）	9（90.0）	23.0（1.3～44.7）	中危组	0.066
极高危组	8（8.6）	8（100.0）	7.0（1.5～12.5）	高危组	0.016
Barcelona-Brno					
低危组	69（70.4）	29（42.0）	未达到		
中危组	15（15.3）	9（60.0）	31.0（14.6～47.4）	低危组	0.099
高危组	14（14.3）	13（92.9）	9.0（1.6～16.3）	中危组	0.012

注：TTFT：诊断到治疗时间

**图1:**
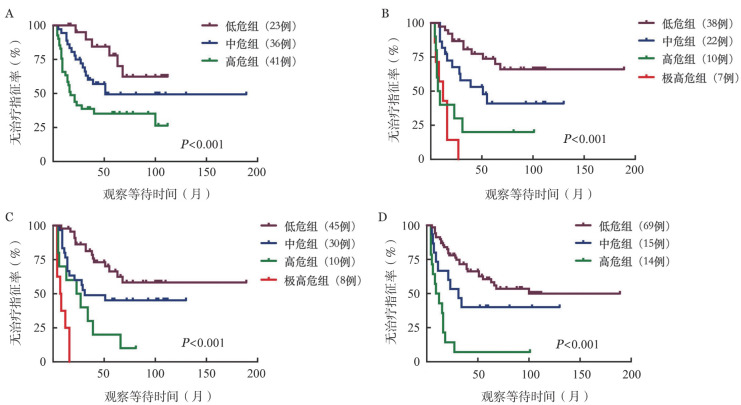
IPS-E（A）、CLL1-PM（B）、CLL-IPI（C）和Barcelona-Brno（D）模型不同危险度慢性淋巴细胞白血病患者的无治疗生存曲线

4. 四种预后模型预测效能评价：采用C-index与AIC进一步对4种预后模型的预测效能进行评价。C-index得分由高到低的模型依次为CLL1-PM（C-index＝0.736，95％ *CI* 0.663～0.808）、CLL-IPI（C-index＝0.722，95％*CI* 0.656～0.787）、IPS-E（C-index＝0.683，95％ *CI* 0.620～0.745）与Barcelona-Brno（C-index＝0.663，95％*CI* 0.595～0.730）。AIC指数由低到高的模型依次为CLL1-PM（AIC＝777）、CLL-IPI（AIC＝933）、Barcelona-Brno（AIC＝986）与IPS-E（AIC＝1004）。

## 讨论

CLL是一类异质性较大的疾病，烷化剂时代Rai和Binet分期被广泛用于判断患者预后，但这两个分期系统仅依靠简单的体格检查和实验室指标。一方面，随着国内CLL诊断水平的进步以及患者诊疗意识的提升，越来越多的CLL患者在诊断时处于疾病早期无症状阶段；另一方面，随着对CLL高危生物学因素的深入研究，IGHV无突变状态、TP53缺失与突变、del（11q）等CLL不良分子遗传学因素的检测广泛应用于临床，为早期筛查无症状CLL患者高危预后因素提供了有利条件。因此，基于早期无症状CLL患者不良生物学预后因素建立预后模型指导患者观察等待期间的临床随访具有很高的临床价值。

本研究基于江苏省人民医院浦口慢淋中心的患者，探索了四种预测无症状Binet A期CLL患者TTFT的预后模型。尽管不同的预后模型纳入的生物学因素各不相同，IGHV突变状态被广泛纳入四个模型中，提示IGHV突变状态对于预测CLL预后有重要价值。Hu等[10]的研究报道证实IGHV无突变患者的TTFT较有突变患者缩短［35.8个月对94.6个月，*HR*＝2.96（95％ *CI* 2.06～4.27），*P*<0.001］。同时，IGHV无突变患者的无进展生存（PFS）时间、OS时间较突变患者缩短，*HR*分别为3.2（95％ *CI* 2.8～3.7，*P*<0.001）和2.4（95％ *CI* 2.0～3.0，*P*<0.001）[11]，证明IGHV突变状态在CLL疾病的发生发展中始终具有关键的预后价值[4],[12]–[15]。本研究团队既往的研究报道提示中国CLL患者的IGHV突变频率较西方患者高，且IGHV使用片段频率与西方患者具有较大差异[16]。而本研究则证实IGHV突变状态在中国CLL患者中具有评估TTFT的重要预后价值（IGHV突变患者和无突变患者的中位TTFT分别为100个月和15个月）。此外，IGHV突变状态在患者疾病进程中保持不变且检测价格较低，有利于患者临床资料回溯，因此推荐CLL患者在诊断初期进行检测。

本研究使用四种预后评分模型对纳入的110例患者进行预后危险度分层。在100例可用于IPS-E预后模型评分的CLL患者中，低危组23例（23.0％）、中危组36例（36.0％）、高危组41例（41.0％）。Condoluci等[8]在提出IPS-E模型时纳入333例CLL患者，低危组171例（51.4％），中危组109例（32.7％），高危组53例（15.9％）。本研究收治的Binet A期CLL患者低危组比例明显较低，而高危组比例明显高于Condoluci等[8]的研究，合理解释了本中心中位随访35个月即有57例（51.8％）患者由于病情进展出现治疗指征。依据IPS-E模型，本中心低危组、中危组和高危组5年内分别有22.1％、50.7％与64.8％的患者启动治疗，与Condoluci等[8]的报道相比，低危组与中危组5年内治疗概率更高，而高危组5年内治疗概率接近。综上，考虑到本研究中高危患者比例较高，且5年内治疗概率也略高于西方人群报道，对于具有明确危险因素的中高危Binet A期CLL患者仍需重视临床定期随访。

IPS-E模型未纳入FISH及二代测序（NGS）等分子遗传学指标，仅需评估IGHV突变状态、淋巴结大小与ALC，通过血常规与临床查体即可进行评价，便于在临床工作中开展。CLL1-PM与CLL-IPI模型均具有较高的C-index与较低的AIC，提示其预测早期无症状CLL患者TTFT的效能较强，两种模型均强调TP53突变或缺失的预后价值。尽管TP53突变或缺失评估CLL患者OS与PFS的不良预后价值受到广泛认可，TP53预测TTFT的价值并不明确。Hu等[10]的队列研究纳入384例初诊未治的CLL患者，发现del（17p）或TP53突变并不是TTFT的独立预后因素，TP53突变或缺失患者的中位TTFT为31.7个月而TP53正常患者的中位TTFT为62.3个月（*P*＝0.500），提示TP53突变或缺失对早期疾病进展的影响有限。与之不同的是，Jeromin等[17]对1160例未治疗CLL患者进行研究，认为TP53突变或缺失是TTFT的独立危险因素（中位TTFT：4.8年对7.5年，*P*＝0.022）。

综上所述，IGHV突变状态对于评估TTFT的预后具有重要价值，且由于其不随疾病进程而变化，推荐在确诊时进行早期检查。四种预后模型均较为成功地对本中心Binet A期CLL患者进行了危险度分层，对中高危患者推荐更积极地进行临床随访，有利于及时发现治疗指征，启动治疗。IPS-E模型是一种相对简单的预后模型，具有很强的临床可及性且检查费用较低。对于有条件接受全面评估的患者，进一步完善FISH与NGS检查有利于更为精准地预测患者的预后。
